# Ppn2 Polyphosphatase Improves the Ability of *S. cerevisiae* to Grow in Mild Alkaline Medium

**DOI:** 10.3390/jof10110797

**Published:** 2024-11-16

**Authors:** Irina A. Eliseeva, Lubov Ryazanova, Larisa Ledova, Anton Zvonarev, Airat Valiakhmetov, Maria Suntsova, Aleksander Modestov, Anton Buzdin, Dmitry N. Lyabin, Ivan V. Kulakovskiy, Tatiana Kulakovskaya

**Affiliations:** 1 Institute of Protein Research, Russian Academy of Sciences, Institutskaya 4, Pushchino 142290, Russia; 2 Federal Research Center “Pushchino Scientific Center for Biological Research of the Russian Academy of Sciences”, Skryabin Institute of Biochemistry and Physiology of Microorganisms, pr. Nauki 5, Pushchino 142290, Russia; 3 Endocrinology Research Center, Dmitriya Ulyanova 11, Moscow 117036, Russia; 4 Institute of Personalized Oncology, I.M. Sechenov First Moscow State Medical University, Bolshaya Pirogovskaya 2 bld. 4, Moscow 119991, Russia; 5 Shemyakin-Ovchinnikov Institute of Bioorganic Chemistry, Miklukho-Maklaya 16/10, Moscow 117997, Russia

**Keywords:** Ppn2, polyphosphatase, KOH, *Saccharomyces cerevisiae*, polyphosphate, CYSTM proteins, RNA-Seq

## Abstract

Inorganic polyphosphates and respective metabolic pathways and enzymes are important factors for yeast active growth in unfavorable conditions. However, particular proteins of polyphosphate metabolism remain poorly explored in this context. Here we report biochemical and transcriptomic characterization of the CRN/PPN2 yeast strain (derived from Ppn1-lacking CRN strain) overexpressing poorly studied Ppn2 polyphosphatase. We showed that Ppn2 overexpression significantly reduced lag phase in the alkaline medium presumably due to the ability of Ppn2 to efficiently hydrolyze inorganic polyphosphates and thus neutralize hydroxide ions in the cell. With RNA-Seq, we compared the molecular phenotypes of CRN/PPN2 and its parent CRN strain grown in YPD or alkaline medium and detected transcriptomic changes induced by Ppn2 overexpression and reflecting the adaptation to alkaline conditions. The core set of upregulated genes included several genes with a previously unknown function. Respective knockout strains (∆*ecm8*, ∆*yol160w*, ∆*cpp3*, ∆*ycr099c*) exhibited defects of growth or cell morphology in the alkaline medium, proving the functional involvement of the respective proteins in sustaining growth in alkaline conditions.

## 1. Introduction

Biochemical and molecular mechanisms underlying the abilities of microbial cells to survive and grow in harsh natural or industrial conditions are crucially important for basic and applied science. Yeast is a classical model eukaryotic microorganism for studying stress adaptation [1,2]. Moreover, stress adaptation is an important factor to consider when using yeast in the biotech industry [3,4] and biomedicine in terms of pathogenic yeast interaction with host cells [5].

Yeast cells possess multiple mechanisms of stress adaptation via stress-triggered signal-transduction pathways, including direct induction of particular condition-specific proteins, accumulation of low-molecular protectants, or adaptive changes in membrane composition of the cell and its organelles [6]. The latter possibility involves significant transcriptomic changes: in yeast, major gene expression changes were observed under various short-term stresses, including abnormal temperature, oxidation, nutrient shift, pH, and osmolarity [7].

Among different stress scenarios, alkalization of culture medium is of particular interest, because it disrupts the electrochemical proton gradient across the yeast plasma membrane. The proton gradient is required for the functioning of all secondary symporters and antiporters which are critical for the uptake of nutrients and ions [8]. Thus, even short-term alkaline stress significantly affects cell state and modulates a substantial number of signaling pathways [9] because it imitates nutrient starvation due to impaired nutrient transport.

Inorganic polyphosphate (polyP), a linear polymer of phosphoric acid, plays an important role in microbial responses to various stresses [10,11,12,13], including alkaline conditions [9]. In chemostat-cultivated *S. cerevisiae*, NMR-observable polyP is degraded to short polymers in response to an increase in external pH [14]. In the case of short-term stress, polyP degradation takes 10–20 min [9,15], suggesting the involvement of specific polyphosphatases which generate phosphate (Pi) as a counterion to neutralize excessive hydroxide ions. Alkali-induced polyP degradation in cells lacking Ppn1 and Ppx1 polyphosphatases is blocked only partially [9,15]. Thus, this process must also involve other proteins of polyP metabolism. In 2017, polyphosphatase Ppn2 was discovered in *S. cerevisiae* [16] but its cellular function remains poorly explored.

Studying Ppn2 function is not trivial, particularly, because Ppn2 overexpression in common strains leads to poor cell survival [17]. Previously, we have introduced a CRN/PPN2 strain with inactivated *PPN1* and overexpressed *PPN2* [17], and characterized by a globally decreased polyP level. An interesting feature of this strain is its ability to grow at higher alkali concentrations compared to its parent CRN [18], providing a convenient model to study the involvement of Ppn2 and polyP metabolism in alkali resistance.

Here, we present a biochemical and transcriptomic characterization of CRN and CRN/PPN2 cells grown under mild alkaline conditions (pH 7–7.5). We highlight Ppn2 as a crucial enzyme that enhances yeast growth in the presence of excess alkali by hydrolyzing cellular polyP and report the functional involvement of several previously unannotated proteins in adaptation to alkaline conditions.

## 2. Materials and Methods

### 2.1. Strains and Growth Conditions

The *S. cerevisiae* CRN/PPN2 strain overexpressing Ppn2 polyphosphatase described in [17] was constructed from the CRN strain (*MATa ade2 his3 ura3 ppn1∆::CgTRP1*) [19]. To construct CRN/PPN2, the parent strain was transfected with the pMB1 expression vector, a derivative of the pRS316 centromeric vector [20], which contains an “expression cassette” with the constitutive TDH3 promoter and PGK terminator.

The BY4741 strain (*MATa his3Δ1 leu2Δ0 met15Δ0 ura3Δ0*) and BY4741Δ*ppn2* knockout mutant strain were obtained from the Dharmacon collection. The BY4742 (*MATα his3Δ1 leu2Δ0 lys2Δ0 ura3Δ0*) and BY4742-derived knockout strains (∆*ecm8*, ∆*adh2*, ∆*yol160w*, ∆*cpp3*, and ∆*ycr099c*) were obtained from the Euroscarf collection.

**Figure 1:**
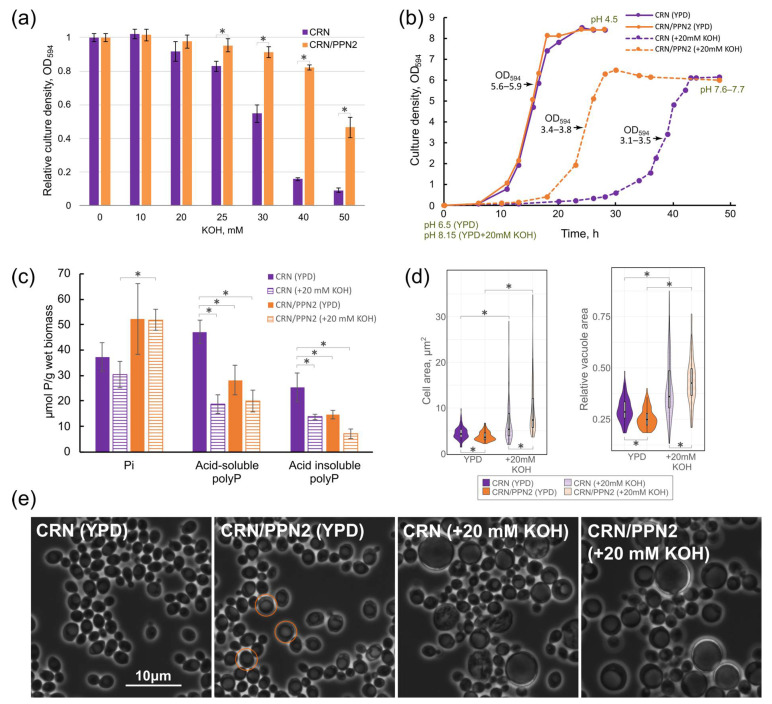
Ppn2 expression affects yeast adaptation to the alkaline medium. (**a**) The effect of alkali concentration on yeast culture density, cultivation for 24 h in immuno-plate in YPD supplemented with varying concentrations of KOH. The relative culture density in 96-well plates was measured at 594 nm. *Y*-axis shows the values normalized to those at 0 mM KOH. Whiskers denote s.d. * *p* < 0.05, two-tailed *t*-test, CRN/PPN2 vs. CRN. (**b**) The growth curves of CRN and CRN/PPN2 strains in the control YPD medium and the YPD medium supplemented with 20 mM KOH. The culture density was measured at 594 nm in a 0.3 cm cuvette. The arrows indicate the growth curve timepoints, at which the biomass was harvested for polyP assay and transcriptome analysis. The ranges of optical densities of the respective cell cultures are labeled directly at the plot. (**c**) Pi, acid-soluble polyP, and acid-insoluble polyP cellular content at the late logarithmic growth stage. The cells were cultivated in 200 mL of control YPD and of YPD supplemented with 20 mM KOH. In control YPD, the cells of both strains were cultivated for 17 h, in YPD supplemented with 20 mM KOH the cells of CRN strain and the cells of CRN/PPN2 strain were cultivated for 42 h and 27 h, respectively. Whiskers: s.d. * *p* < 0.05 two-tailed *t*-test. (**d**) Distribution of the absolute cell area and the relative vacuole area (normalized to the cell area) of CRN and CRN/PPN2 cells. In control YPD, the cells of both strains were cultivated for 17 h; in YPD supplemented with 20 mM KOH, the cells of CRN and CRN/PPN2 strains were cultivated for 42 h and 27 h, respectively. * *p* < 0.01, unpaired two-samples Wilcoxon test. (**e**) The phase contrast microphotographs of CRN and CRN/PPN2 cells. Red circles pinpoint the cells of CRN/PPN2 strain having a round shape and enlarged vacuoles in the control YPD medium.

The cells were cultivated in flasks with 100–200 mL of culture medium at 29 °C and 145 rpm in YPD containing 2% glucose, 2% peptone (Peptone from meat enzymatic digest, Fluka, Sigma-Aldrich, St. Louis, MO, USA), 1% yeast extract (CONDA/Pronadisa, Madrid, Spain). In our case, the base YPD medium contained 3 mM Pi. Alkaline media contained KOH in different concentrations (as indicated in the text). The culture density (Figure 1b) was measured with the Unico-2100 spectrophotometer (Unico, Shanghai, China) at 594 nm in a 0.3 cm cuvette.

The cells were cultivated until reaching the late logarithmic growth stage. In the control YPD medium, the cells were cultivated for 16 h. In YPD supplemented with 20 mM KOH the cells of CRN and CRN/PPN2, strains were cultivated for 40 h and 25 h, respectively. The biomass samples were separated from the culture medium by centrifugation at 5000× *g* for 15 min. Next, the biomass was washed with MiliQ water, followed by another centrifugation at 5000× *g* for 15 min. Washing and centrifugation were repeated twice. The cells CRN/PPN2 strain grown in the alkaline medium were lysed in distilled water and therefore these cells were washed in MiliQ water with 0.2 mM sorbitol.

### 2.2. PolyP Extraction, Quantification, and Chain Length Analysis

The biochemical assays were performed in triplicates. Acid-soluble polyP was extracted from biomass with 0.5 M HClO_4_ at 0 °C as described earlier [21]. PolyP content in the extracts was estimated by phosphate releasing after 20 min treatment with 0.5 M HClO_4_ at 90 °C. The amount of acid-insoluble polyP remaining in the biomass was estimated by the phosphate release after 20 min treatment with 0.5 M HClO_4_ at 100 °C [21]. Phosphate quantification was performed using a molybdenum blue colorimetric method as in [22]. 1 mL of 10% aqueous solution of ascorbic acid was added to 100 mL of aqueous solution containing 2% H_2_SO_4_, 0.5% sodium dodecyl sulfate, and 0.5% ammonium molybdate. 2 mL of the mixture was added to 1 mL of the sample. The absorbance was measured at 750 nm after 10 min incubation at 30 °C. The amount of polyP was normalized to the wet weight of biomass obtained under the same centrifugation conditions [21].

For polyP chain length analysis (Figure S1), the polyPs were extracted as described earlier [23]. To obtain the fraction of the shortest chain length (polyP1), the biomass was treated twice with 0.5 M HClO_4_ at 0 °C for 20 min with stirring. The medium-sized polyPs (polyP2) were extracted from the remaining biomass as follows: after the separation of the supernatant, the biomass was treated twice with saturated NaClO_4_ at 0 °C for 30 min; the supernatant was collected after centrifugation. Finally, the long-chain polyP3 was extracted with 0.1 mM NaOH (pH 10), at 0 °C for 20 min. The polyP1, polyP2, and polyP3 samples were precipitated with a saturated solution of Ba(NO_3_)_2_ and re-solved by treating with Dowex AG 50Wx8 (Sigma-Aldrich) ion exchange resin in (NH_4_)^+^ form [23].

The chain length of each polyP fraction (polyP1, polyP2, and polyP3) was assessed by electrophoresis using samples with equal amounts of polyP (measured by the total phosphorus content). The electrophoresis was performed as described previously [24] in 24% polyacrylamide gels with 7 M urea; commercial polyP15, polyP25, and polyP75 (Sigma-Aldrich) and polyP188 (Monsanto) were used as the chain length markers (the numbers indicate the average amount of phosphate residues in the polyP chain). PolyPs were visualized by staining the gels with the toluidine blue.

### 2.3. Cell Counting and Assessing Yeast Growth in Alkaline Conditions

The cell concentration in culture samples was measured by flow cytometry with the NovoCyte Flow cytometer (Agilent, Santa Clara, CA, USA). Yeast samples normalized by cell concentration (0.5·10^7^ cell/mL) were added to sterile 96-well plates. Each well contained 0.2 mL of the YPD medium supplemented with different concentrations of KOH. The cells were cultured for 24 h. Afterward, the optical density was measured at 594 nm with an EFOS photometer (Sapphire, Moscow, Russia), Figure 1a and Figure S2.

### 2.4. Cell and Vacuole Size Estimation

For Figure 1d, we estimated cell and vacuole size distribution from 100 to 110 cells present in 1–2 micrographs for each strain using ImageJ [25].

### 2.5. Light Microscopy

The microphotographs were obtained with AXIO Imager A1 ZEISS (Carl Zeiss AG, Oberkochen, Germany).

### 2.6. RNA Extraction and High-Throughput Sequencing

The cells were cultivated for 16 h (control medium, both strains), 40 h (20 mM KOH, CRN), and 25 h (20 mM KOH, CRN/PPN2). Two replicates were performed for each combination of strain and growth conditions, for 8 samples in total.

**Treatment protocol.** The biomass samples were water-washed in triplicate. Samples grown in the control medium and the CRN samples grown in the alkaline medium were frozen at −70 °C, homogenized with French press, and suspended in Trizol LS. The samples of CRN/PPN2 strain grown in the alkaline medium were lysed with Trizol LS immediately.

**RNA extraction protocol and sequencing library preparation.** Total RNA was extracted with Trizol LS (Thermo Fisher Scientific, Waltham, MA, USA) and then purified on Direct-zol RNA Microprep columns (Zymo Research, Irvine, CA, USA). Libraries were prepared according to the manufacturer’s protocol without any modifications using the NEBNext Ultra Directional RNA Library Prep Kit for Illumina (NEB, Ipswich, MA, USA) and NEBNext Poly(A) mRNA Magnetic Isolation Module (NEB, Ipswich, MA, USA) for rRNA depletion. The resulting libraries were sequenced with Illumina NextSeq 500 (Illumina, San Diego, CA, USA).

### 2.7. Bioinformatics Analysis

The raw read quality was assessed with FastQC [26]. The sequencing depth per sample was around 4 to 6 mln except CRN/PPN2 (1 to 2 mln), and 80–90% of the reads were mapped uniquely using STAR v.2.7.6a (default parameters) [27] against Ensemble *S. cerevisiae* R64-1-1 genome assembly (Ensembl R-64-1-1.107 transcript annotation). Gene-level read counts were obtained with STAR (--*quantMode GeneCounts*). The resulting gene lists were filtered to contain only the genes reaching at least 10 counts per million in at least two of the eight libraries. The first and the second replicates of all samples were made in two independent sample preparation and sequencing batches, and the ComBat-seq function from sva (v.3.42.0) [28] was used for batch correction. Read counts were normalized using the edgeR (v.3.36.0) [29] TMM approach. A principal component analysis of the normalized and log-transformed matrices and other statistical tests were performed in the R environment v.4.1.3. Differential expression was assessed with the edgeR generalized linear model, the genes were considered differentially expressed if passed 5% FDR (false discovery rate, Benjamini–Hochberg correction for multiple tested genes). Gene set enrichment analysis (GSEA) was performed with the FGSEA R package v.1.20.0 [30]. Gene ontology subset (GO SLIM) annotation for GSEA was retrieved from the Saccharomyces Genome Database [31]. Batch-corrected gene-level read counts, differential gene expression data, and GSEA results are provided in Table S1.

## 3. Results

### 3.1. PPN2 Expression Enhances Alkali Resistance

CRN/PPN2 strain overexpressing Ppn2 polyphosphatase is a new model to study the involvement of phosphorus metabolism in alkali resistance: this strain is more resistant to alkali compared to parent strain CRN in the immune plate test with different KOH concentrations (Figure 1a, Table S2). The dependency on Ppn2 was also observed in the comparison of a standard BY4741 versus BY4741∆*ppn2*, where the lack of Ppn2 led to decreased alkali resistance (Figure S2, Table S2).

To reveal the dynamics of cell growth in the medium supplemented with 20 mM KOH, we cultivated CRN and CRN/PPN2 strains until the stationary growth stage (Figure 1b). We measured the initial and the final pH values: pH started at 6.5 in the control YPD medium and decreased to 4.5 for both strains at the stationary growth stage. In the case of the alkaline medium (YPD medium with 20 mM KOH), the starting pH was 8.15, and it decreased only slightly to 7.6–7.7 at the stationary growth stage for both strains. Thus, under these conditions, the cells of both strains grew in the weak alkaline medium. Usually, the yeast acidify the medium to pH 4–4.5, which is necessary to maintain the proton gradient on the plasma membrane [32]. We hypothesize that, in our conditions at pH over 7, the proton gradient was significantly decreased. Both strains showed lower culture density and longer lag-phase in the alkaline medium, but CRN/PPN2 had a shorter lag phase compared to CRN; there were no differences in the control YPD medium (Figure 1b). Alkalization of the medium was shown to trigger the degradation of polyP in the first 10–20 min [15]. In our case, the level of acid-soluble short-chain polyP and acid-insoluble long-chain polyP in the cells of CRN at the late logarithmic stage was nearly halved in the alkaline medium (Figure 1c), and this decrease was observed for CRN/PPN2 in both growth conditions. To estimate the polyP fragmentation, we analyzed three polyP fractions comprising more than 90% of the total cellular polyP content [23] (see Figure S1). The average polyP chain length in CRN/PPN2 (YPD) was shorter than that of CRN, as expected from Ppn2 overexpression. In the alkaline medium, the average polyP chain length was shortened for both strains. The polyP chain shortening effect was specifically exhibited in CRN/PPN2, for which the staining had very low intensity despite the amount of total phosphorus in all samples being identical. It is known that very short polyPs are poorly stained by toluidine blue and thus remain invisible in electropherograms [33] (such as shown in Figure S1). Thus, reduced staining of CRN/PPN2 samples indicates a high level of polyP fragmentation. Although Ppn2 does not possess exopolyphosphatase activity, CRN/PPN2 in the alkaline medium showed a high Pi level (Figure 1c), likely to be due to more efficient hydrolysis of shorter polyP chains by Ppx1 [34]. Apparently, the extra Pi and short-chain polyP act as counter-ions allowing for better cell growth in the alkaline medium.

The CRN and CRN/PPN2 cell populations contained larger cells with specifically enlarged vacuoles when cultivated in the alkaline medium (Figure 1d,e). This is consistent with the role of vacuoles in maintaining cellular pH [15]. The increase in vacuole size can be explained by the excess phosphate localized in these organelles [35]. In the YPD medium, cell populations of CRN/PPN2 strain already included round-shaped cells with enlarged vacuoles (Figure 1d,e).

Thus, with respect to polyP level, chain length, and cell morphology, the CRN/PPN2 strain cultivated in YPD has some features characteristic of growth in the alkaline medium. We suggest that Ppn2 polyphosphatase is directly involved in alkaline adaptation through polyP hydrolysis.

### 3.2. Transcriptome Profiling Reveals the Details of the Interplay Between Phosphate Metabolism and Adaptation to Alkaline Conditions

#### 3.2.1. Ppn2 Overexpression Activates General Stress Response

**Figure 2:**
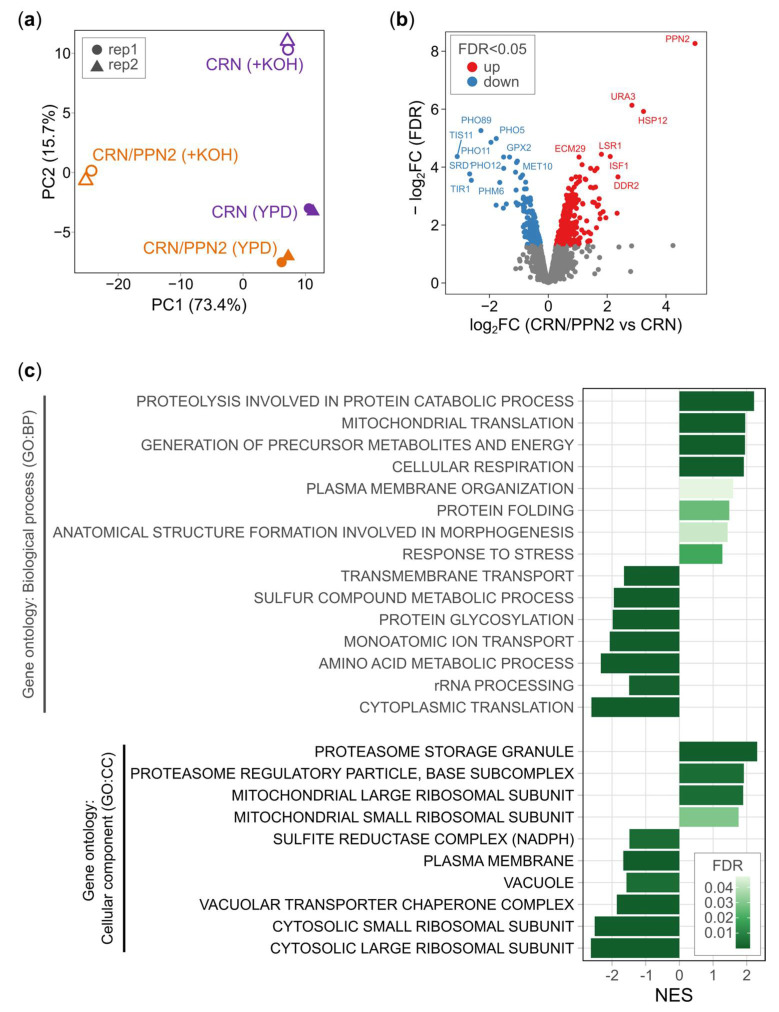
Comparative transcriptomic characterization of CRN/PPN2 strain. (**a**) Principal component analysis (PCA) of normalized and batch-corrected RNA-Seq data. The percentage of variation explained by a particular principal component (PC) is indicated in the axis label. Point shape and coloring are consistent with the strain and cultivation condition. (**b**) Volcano plot of transcriptomic changes in CRN/PPN2 versus CRN, both cultivated in normal conditions (YDP); genes with adjusted *p*-value (FDR) < 0.05 are colored, genes with FDR ≥ 0.05 are shown in grey. (**c**) Gene set enrichment analysis (GSEA) of changes in CRN/PPN2 versus CRN, cultivated in normal conditions (YDP). Genes were sorted by signed adjusted *p*-value. The selected significantly enriched gene ontology terms are shown; NES—Normalized Enrichment Score.

To study the adaptation to alkaline conditions on the level of gene expression, we performed RNA sequencing for CRN and CRN/PPN2 cells in normal YPD (16 h) and YPD supplemented with 20 mM KOH (40 h for CRN and 25 h for CRN/PPN2) until the late logarithmic stage. Principal component analysis of batch-corrected gene-level read counts revealed good concordance between replicates and clear separation of strains and cultivation conditions (Figure 2a). Particularly, CRN/PPN2 (+KOH) is positioned far apart from other samples along the principal component 1, which explains more than 70% of the total variance.

To identify specific changes, we performed differential gene expression analysis. The comparison of CRN/PPN2 vs. CRN under normal conditions showed that nearly 10% (1.2%) of genes were differentially expressed at 5% FDR (5% FDR and |Log_2_ Fold Change| > 1) (Figure 2b). For upregulated genes, the maximal change, as expected, was observed for PPN2 (on the top-right of the volcano plot). The most significantly downregulated genes included several members of the PHO (Phosphate-responsive signal transduction) pathway such as Pi transporter gene *PHO89*, *PHO5* encoding acid phosphatase, and *PHM6* encoding cytoplasmic membrane protein.

To further explore the transcriptomic profile of CRN/PPN2, we performed gene set enrichment analysis with GO (gene ontology) Biological Process (GO BP) and Cellular Compartment (GO CC) annotations [31]. Compared to CRN, CRN/PPN2 exhibited upregulation of genes related to proteasome, proteolysis, morphogenesis, and general stress response (Figure 2c). Plasma membrane terms demonstrated significant enrichment considering both up- and downregulated genes, suggesting the membrane undergoes major restructuring. Among downregulated terms, we focused our attention on transmembrane transport and vacuole proteins, including the VTC (vacuolar transporter chaperone) complex. Altogether, these observations indicate the general stress state of the CRN/PPN2 cells, although it does not affect their growth dynamics in the normal medium.

A special case is the activation of cellular respiration and mitochondrial translation genes, in parallel to the downregulation of cytoplasmic translation. The latter is likely also to be related to the general stress response. However, we cannot give a straightforward interpretation of the enhanced expression of mRNAs associated with mitochondrial translation and respiration, except for possible response to acidification of the cytoplasm due to phosphate excess.

#### 3.2.2. CRN/PPN2 Exhibits Enhanced Differential Expression in the Alkaline Medium

**Figure 3:**
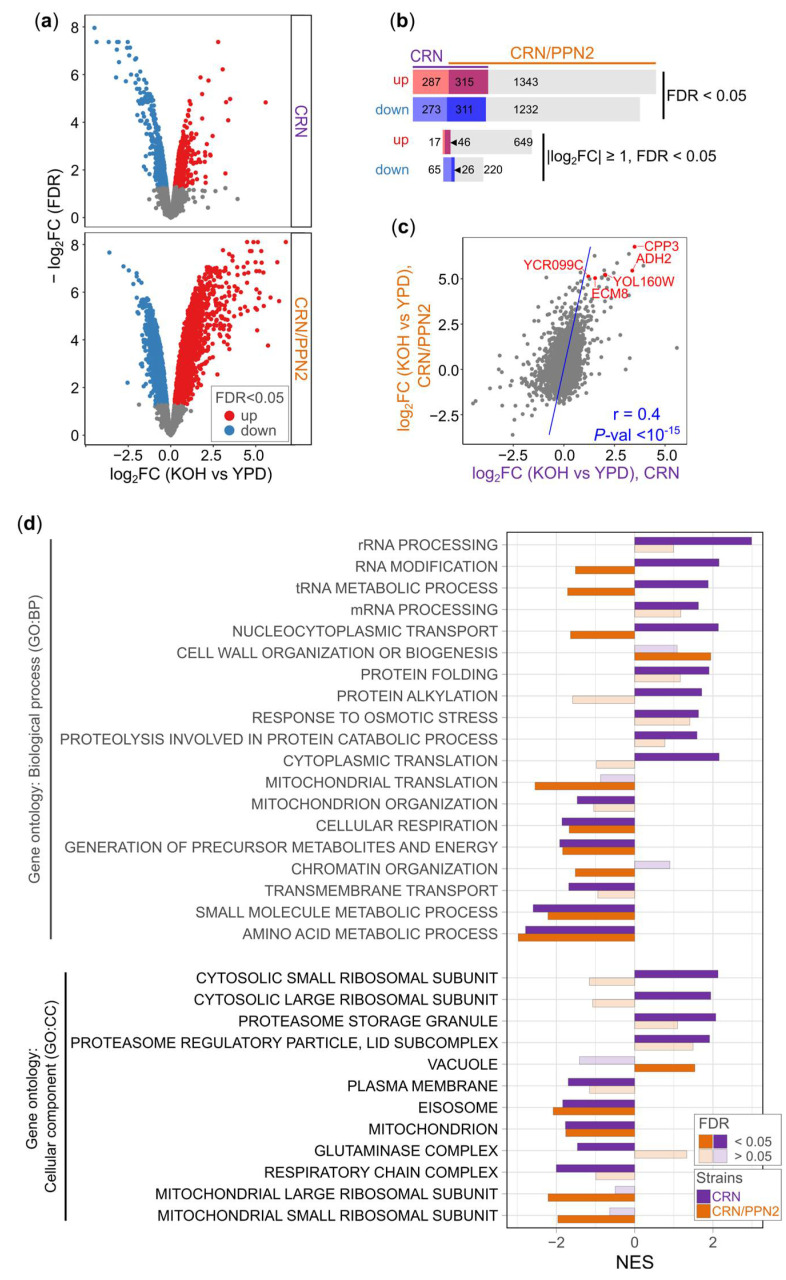
Yeast transcriptome changes in adaptation to alkaline conditions. (**a**) Volcano plots of transcriptomic differences between cultivation conditions (YPD supplemented with 20 mM KOH versus normal conditions, YPD) in CRN (upper panel) and CRN/PPN2 (the lower panel) cells. Genes with adjusted *p*-value (FDR) < 0.05 are colored, genes with FDR ≥ 0.05 are shown in grey. (**b**) Number of shared and unique significantly up- and down-regulated genes in KOH versus normal conditions in CRN (left) and CRN/PPN2 (right). The thresholds are shown. (**c**) Scatter plot shows the correlation of changes in gene expression between different cultivation conditions (YPD supplemented with 20 mM KOH versus normal conditions, YDP) in CRN (*x*-axis) and CRN/PPN2 (*y*-axis). Pearson’s CC and *p*-value are shown. The genes selected for the consequent analysis of mutant strains are labeled in red. (**d**) Gene set enrichment analysis (GSEA) of gene expression changes between cultivation conditions (YPD supplemented with 20 mM KOH versus normal conditions, YDP). Genes were sorted by signed adjusted *p*-value. The selected significantly enriched KEGG pathways are shown; NES—Normalized Enrichment Score.

To explain at the molecular level the enhanced ability of CRN/PPN2 to grow under alkaline conditions, we performed differential gene expression analysis for CRN and CRN/PPN2 separately, comparing the transcriptomes obtained in YPD versus YPD +20 mM KOH. CRN/PPN2 was strikingly powerful in adapting its gene expression: expression of nearly 60% (17.5%) genes changed significantly at 5% FDR (5% FDR and |Log_2_ Fold change| > 1), while only 22% (2.8%) genes were affected in CRN in cells grown in the alkaline medium (Figure 3a,b).

Interestingly, for both strains, the transcriptomic changes in alkaline conditions were different from those reported in multiple previous transcriptomic studies of alkali stress in *S. cerevisiae* [36,37,38,39,40,41] (Figure S3). We observed very low positive (~0.1 or lower (for CRN/PPN2) and negative (for CRN) Pearson correlations of gene-level fold changes under alkali stress versus alkali adaptation. We consider these differences to be caused by different time scales: short-term stress (from 5 min to 2 h, published data) and long-term growth (20–40 h, our study) yield distinctly different physiological states. Of note, CRN/PPN2 adaptation is more similar to that observed for the usual yeast strains under alkali stress in previous studies, likely to be due to CRN being initially hampered by lack of Ppn1.

Despite striking differences in the scale of transcriptomic changes under alkaline conditions in CRN versus CRN/PPN2, the general changes were, in fact, concordant: nearly half of the genes differentially expressed in CRN were also differentially expressed in CRN/PPN2 (Figure 3b) and overall Pearson’s correlation was ~0.4 (Figure 3c). Further, gene ontology enrichment also yielded similar results (Figure 3d), although some gene ontology terms were statistically significant only for CRN/PPN2.

The growth in alkaline conditions led to decreased expression of genes involved in mitochondrial function, transmembrane transport, plasma membrane, and general metabolism. These pathways are not specific for alkali adaptation, but, as in the CRN/PPN2 versus CRN comparison, reflect general stress response [41,42]. Interestingly, there were only a few GO terms upregulated exclusively in CRN/PPN2: cell wall organization (GO BP) and vacuole (GO CC). CRN/PPN2 cells are easily lysed and upregulation of cell wall organization genes likely reflects compensatory mechanisms necessary to maintain cell wall nativity in alkaline conditions; in addition, in the alkaline medium, the vacuoles of CRN/PPN2 cells are morphologically larger than those of CRN. Thus, both effects are connected with the visible changes in cell morphology. The gene groups uniquely upregulated in CRN encode components of protein translation machinery, yet this observation remains hard to interpret given that the gene expression changes for this strain were less pronounced.

**Figure 4:**
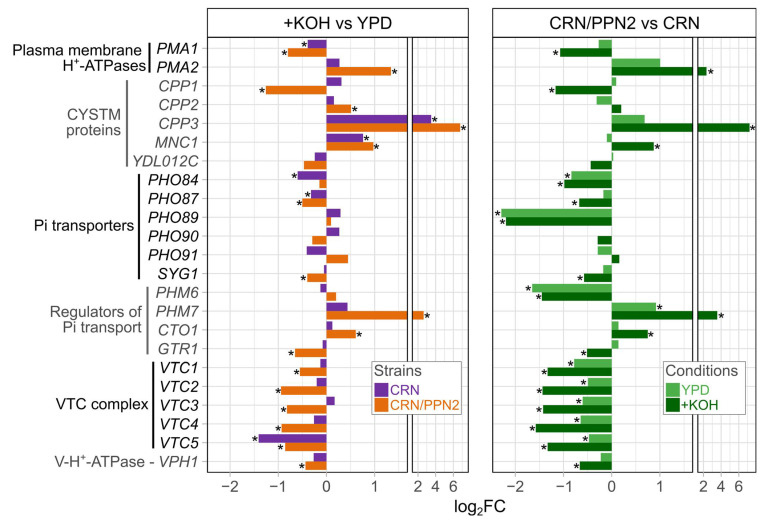
Expression changes of selected gene groups: genes directly involved in maintaining the proton gradient on the plasma membrane, phosphate ion transport (GO:0006817), polyphosphate metabolic process (GO:0006797), and encoding CYSTM proteins. (**Left panel**) changes in expression in 20 mM KOH versus YDP. (**Right panel**) changes in CRN/PPN2 versus CRN. * FDR < 0.05.

We consider CRN/PPN2 as a model of impaired phosphorus metabolism. Thus, we focused our attention on particular functional groups of the respective genes (phosphate ion transport, GO:0006817, and polyphosphate metabolic process GO:0006797) and their changes in alkaline conditions (Figure 4): Pi transporters, Pi transport regulators, VTC complex subunits, and VPH1, subunit a of the vacuolar-ATPase V0 domain. In addition, we considered plasma-membrane H^+^-ATPases as they are directly involved in maintaining the proton gradient on the plasma membrane [43], and CYSTM (cysteine-rich transmembrane module) proteins which are involved in diverse stress response and adaptation to harsh growth conditions [44,45].

Strikingly, three functional groups (VTC complex, responsible for polyP biosynthesis, CYSTM proteins, and plasma membrane H^+^-ATPases) exhibited fully concordant changes in expression when comparing (1) alkali versus normal conditions and (2) CRN/PPN2 versus CRN (Figure 4). In regard to the expression of these genes, CRN/PPN2 is preadapted to growth in the KOH medium. Two other gene groups, Pi transporters and Pi transport regulators, also displayed generally concordant changes. However, there were two strongly discordant genes, *PHO89* and *PHM6*: they were nearly neutral in KOH vs. YPD comparison but strongly downregulated in CRN/PPN2 versus CRN (see also Figure 2b).

### 3.3. Mutant Strains with Different Adaptability to Alkali

**Figure 5:**
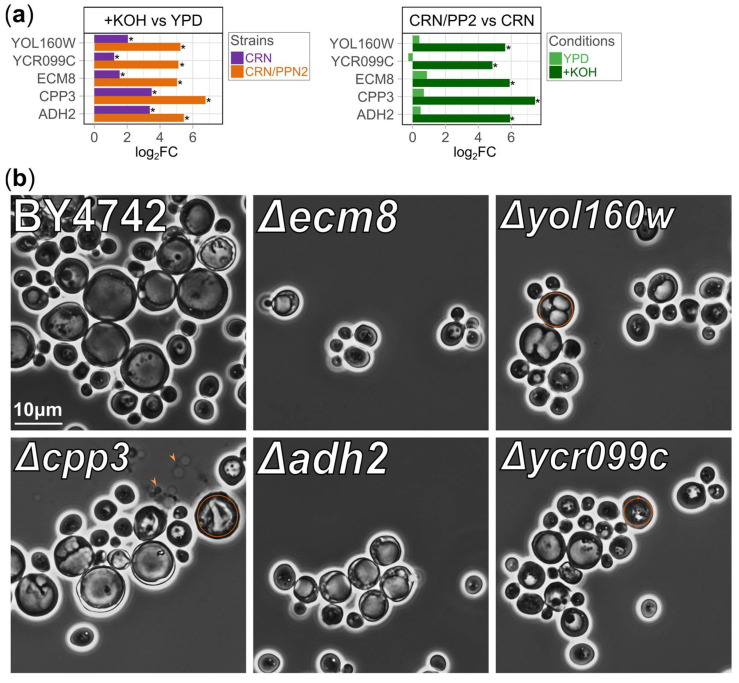
Cell morphology changes of yeast knockout strains grown in the alkaline medium. (**a**) Upregulated expression of selected poorly annotated genes in CRN and CRN/PPN2 cells grown in the alkaline medium (YPD supplemented with 20 mM KOH). * FDR < 0.05. (**b**) Phase contrast microphotographs of various strains of *S. cerevisiae* cultivated in YPD supplemented with 40 mM KOH. The orange circles pinpoint important morphological changes. The orange arrows indicate cell lysis.

Gene set enrichment analysis did not reveal easily interpretable pathways upregulated in yeast grown in the alkaline medium except for general stress response. We hypothesized that adaptation to alkaline conditions might involve less commonly studied genes. From those, we selected five that were highly upregulated in the alkaline medium in CRN and even higher in CRN/PPN2, and, moreover, upregulated in CRN/PPN2 compared to CRN in YPD (Figure 5a), likely contributing to the pre-adaptation of CRN/PPN2 cells to the alkaline medium. This special group of interest (Figure 5a) comprised three poorly annotated genes (*ECM8*, *YOL160W*, *YCR099C*) as well as *ADH2* (alcohol dehydrogenase 2, Glucose-repressible alcohol dehydrogenase II) [46] and *CPP3* (C-terminally Palmitoylated Protein) [47]. Prioritizing these genes became possible owing to unique features of CRN/PPN2 and large-scale gene expression changes in long-term growth in alkaline conditions, which yielded a transcriptome profile distinct from the usually studied short-term stress (Figure S3). Interestingly, *ADH2* and *CPP3* are also strongly upregulated at manganese excess [48].

To assess the functional importance of these genes for efficient growth and maintenance of the cell morphology in the alkaline medium, we used knockout strains ∆*ecm8*, ∆*adh2*, ∆*yol160w*, ∆*cpp3*, and ∆*ycr099c* available from the Euroscarf collection and derived from BY4742 (*MATα his3Δ1 leu2Δ0 lys2Δ0 ura3Δ0*). To study the changes in the alkaline medium, YPD was supplemented with 40 mM KOH, as BY4742 cells are resistant to 20 mM KOH used for CRN and CRN/PPN2. In these alkaline conditions, BY4742 shows the same morphology abnormalities as CRN with 20 mM KOH: large rounded cells with enlarged vacuoles (Figure 1e and Figure 5b).

When cultivated in control YPD, the cells of all strains had normal morphology and there were no differences in cell concentration between strains after 24 h of cultivation (Figure S4). When cultivated in the alkaline medium, the Δ*ecm8* strain yielded significantly reduced cell concentration, although the other strains showed the same or even higher cell concentration compared to BY4742 (Figure S4).

In terms of cell morphology, the cultivation in the alkaline medium led to diverse effects. (1) There were no cells with enlarged vacuoles for ∆*ecm8*. The lack of enlarged vacuoles may explain why these knockouts cannot efficiently grow in alkaline conditions, as vacuole enlargement decreases vacuole lumen alkalization by water dilution. (2) Vacuoles were fragmented in Δ*yol160w* cells, although the mechanism remains unclear. (3) Δ*cpp3* cell population exhibited cell lysis and included multiple cells with destroyed vacuoles. Cpp3 is attached to the plasma and vacuolar membranes via covalently attached palmitic acid residue [47], and we hypothesize that its CYSTM domain is exposed to the cytoplasm and vacuolar lumen, forming clusters to strengthen the membranes. (4) ∆*adh2* cells had no special features and were indistinguishable from those of BY4742. (5) ∆*ycr099c* cells had severely damaged vacuoles and vacuoles with multiple dark inclusions of unknown nature. All in all, knockout cells grown in the alkaline medium exhibited diverse abnormalities of vacuoles.

We conclude that *ADH2* is not involved in adaptation to alkaline medium, despite being one of the top upregulated genes. Instead, *ECM8* is crucial for survival in the alkaline medium, although the mechanism remains unknown. *YOL160W*, *YCR099C*, and *CPP3* contribute to the vacuole reorganization necessary for effective adaptation to alkaline conditions.

## 4. Discussion

Unfavorable environmental conditions and stress factors, such as oxidants, thermal shock, nitrogen depletion, amino acid starvation, acidification, or alkalization of the medium [7,49,50,51,52], and the elevated concentrations of heavy metal ions [53] induce significant changes in yeast cell growth, metabolism, morphology, and gene expression. In fact, the majority of differentially expressed genes that we detected in our study are not specific for growth in the alkaline medium (pH 7.5) but reflect general suppression of cell growth: elongated lag-phase, lowered cell concentration, and altered cell morphology (enlarged vacuoles, Figure 1c,e), and many of these effects were described previously for other stress conditions. Particularly, downregulation of ribosome biogenesis and cytoplasmic translation were detected under short-term alkaline stress [41]; while proteasome genes upregulation was observed in heat stress [54], and proteasome activity was shown to increase under nitrogen starvation [55] and freezing [56]. It is well known, that protein activity and stability are pH-dependent [57]. Further, mild alkaline treatment can be used for protein extraction from yeast [58]. We hypothesize that yeast growing in low-alkaline pH conditions may also have higher intracellular pH levels, provoking proteotoxic stress from improperly folded proteins and activating the ubiquitin–proteasome system [59].

The transcriptional programs activated in response to alkaline stress or long-term growth in an alkaline medium are different (Figure S3). However, particular functional gene groups (Figure 4, Table S1) deserve special attention. First of all, *RIM21* encoding polytopic plasma membrane protein that senses external alkalization is among the key genes activated in alkali stress response [7,60,61]. However, in our setup, *RIM21* was upregulated only in CRN/PPN2 [60,62]. The gene encoding its downstream target *Rim101* (a transcription factor that regulates gene expression resulting in the expression of cell wall proteins, secreted enzymes, and proteins required for ion homeostasis) is upregulated in response to short-term alkali exposure [7,60,61] but, in our setup, it expression was stable.

It is known that alkaline stress and Pi starvation [9] induce concordant changes in genes encoding Pi transporters: H^+^/Pi symporters *PHO87* and *PHO90* are downregulated, while *PHO84* and Na/Pi symporter *PHO89* are upregulated [7,9,63]. In this context, the similarity between Pi starvation and alkaline stress is explained by the following considerations [9]. Low-affinity Pi transporters (Pho87, Pho90) and major high-affinity Pi transporter, Pho84, cannot perform H^+^/Pi symport [64,65] when the electrochemical H^+^ gradient decreases, which occurs directly in case of alkalization of the extracellular medium or, indirectly, due to Pi starvation-induced drop in ATP concentration. Yet, in our setup with continuous growth in the alkaline medium, *PHO84* and *PHO87* are downregulated, while *PHO89* and *PHO90* expression remains mostly unchanged (Figure 4). We consider this a direct consequence of polyP hydrolysis by Ppn2 yielding shorter polyP chains, which are then used as the source of extra Pi (Figure 1d) by Ppx1 or pyrophosphatases.

Phm6 and Phm7 are membrane proteins expressed after phosphate starvation and responsible for the overaccumulation of phosphate [66]. *PHM7,* but not *PHM6,* is upregulated in the case of growth in alkaline media (Figure 4), similar to their changes under manganese excess [67]. Yet, as their functions are not known in detail, it remains hard to interpret this effect in the context of phosphate uptake. Particularly, they behave similarly under very short-term alkali stress but are both downregulated in the case of 1-h exposure [41].

The next crucial part of phosphorus homeostasis is the polyP-synthesizing VTC complex [68]. Similar to *PHO89*, genes encoding VTC subunits *VTC1* and *VTC4* are upregulated in both short-term alkali stress and Pi starvation [9,69], but genes of all VTC subunits are downregulated in our setup. VTC works in tandem with V-ATPase [70,71], and *VPH1* encoding one of the V-ATPase subunits is also significantly downregulated. Altogether this means that phosphate consumption for polyP synthesis decreases significantly in alkaline conditions, thereby lowering the demand of cellular phosphate for polyP synthesis and allowing for cell survival in spite of downregulated phosphate transport.

The primary function of V-ATPase is pumping H^+^ from the cytoplasm to the vacuolar lumen [72]. *VPH1* downregulation must decrease V-ATPase activity and, consequently, lead to acidification of the cytoplasm in alkaline conditions. Another protein pumping H^+^ from the cytoplasm is Pma1 ATPase of the plasma membrane [43], and it was also downregulated in alkaline conditions (Figure 4), additionally contributing to cytoplasm acidification. Another plasma membrane ATPase *PMA2* is strongly upregulated, although it is unlikely to substitute for the lack of Pma1 as *PMA2* mRNA abundance is an order of magnitude lower than that of *PMA1*. Of note, *PMA2* upregulation was also observed at the stationary growth [73] and in manganese adaptation [48].

CYSTM proteins form a less-studied group of stress response and adaptation factors [44]. These proteins possess conserved cysteine-rich domains and are anchored in membranes by covalently attached palmitic acid [47]. *S. cerevisiae* genome annotation includes five CYSTM proteins: Cpp1, Cpp2, Cpp3, Mnc1, Ydl012c. Of those, genes of two orthologs, Mnc1 and Cpp3, are highly upregulated in the alkaline medium. Previously, we had shown *MNC1* involvement in manganese adaptation [48] and, in this study, we reported its upregulation in the alkaline medium on the transcriptomic level (Figure 4). However, microphotographs of yeast carrying GFP-MNC1 fusions do not show a higher abundance of the protein, because GFP fluorescence under alkaline conditions was quite low [45]. On the contrary, in alkaline conditions, Cpp3 was upregulated both on the transcriptomic (this study) and proteomic level [45]. Further, in alkaline conditions, ∆*cpp3* cells exhibit cell lysis (Figure 5b).

We have also detected the major alkali-induced upregulation of *ADH2*, which is also upregulated under manganese excess [48]. Yet the growth and cell state of the ∆*adh2* knockout strain were the same as those of the parent strain under both alkaline conditions and manganese excess. This gene is usually expressed under glucose depletion and supposedly is not responsible for any adaptive changes but is associated with the slowdown in cell growth.

We formulate the following hypothesis for the improved ability of CRN/PPN2 to adapt to alkaline conditions. (1) Ppn2 can efficiently produce short polyP and Pi as counterions working in tandem with Ppx1 and pyrophosphatases, allowing neutralization of excess OH^-^ in cytoplasm and vacuole. Ppn2 is usually localized in the vacuole [16], although CRN/PPN2, in addition, has Ppn2 in the cytoplasm [18]. (2) Ppn2 overproduction induces transcriptomic changes which are partly pre-adaptive for growth in alkaline conditions. The induction of such a state is probably not a direct result of decreased polyP level: the ∆*vtc4* knockout strain with decreased total polyP level has a lower capability to grow in the alkaline medium [74]. Of note, an important feature of CRN/PPN2 cells is the increased short-chain polyP fraction, while ∆*vtc4* cells lack short-chain polyP, which we consider an important counter-ion for alkali.

**Figure 6:**
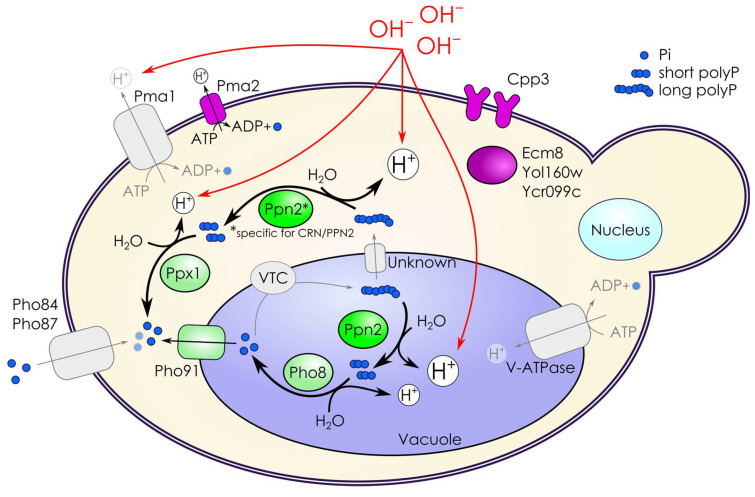
Model of Ppn2 involvement in adaptation of CRN/PPN2 cells to mild alkaline medium. Vacuole alkalinization increases Ppn2 efficacy in producing short-chain from long-chain polyPs yielding excess Pi as counter-ion for both CRN and CRN/PPN2. The same process in the cytoplasm is specific for CRN/PPN2. The details are given in the text.

Summing up our findings, we formulated the following model of Ppn2’s role in alkaline adaptation (Figure 6). Alkaline medium increases OH^-^ intake in cytoplasm and vacuole. The downregulation of Pma1 and V-ATPase decreases proton pumping from the cytoplasm, which compensates for excessive OH^-^. This decrease cannot be fully compensated for by Pma2 upregulation due to its low absolute expression. The reduced proton gradient decreases Pi intake by Pho84 and Pho87, and this effect is further enhanced by lowered expression of the respective genes. This results in a major decrease in the cellular phosphate intake. However, genes encoding VTC complex and V-ATPase subunits are downregulated, limiting Pi usage for polyP synthesis. Ppn2 enzymatic activity has a neutral pH optimum. Usually, vacuoles have acidic pH and alkalization of vacuolar lumen improves Ppn2 efficacy in digesting long-chain polyPs into short-chain polyPs. In turn, short-chain polyPs serve as counter-ions and are further digested by alkaline phosphatase Pho8 producing Pi, which Pho91 then transports to cytoplasm. Uncharacterized proteins Cpp3, Ecm8, Yol160w, and Ycr099c participate in vacuole function and contribute to its normal morphology through unknown mechanisms. Specifically for CRN/PPN2, the cytoplasmic activity of Ppn2 generates extra short-chain polyPs, which are hydrolyzed by Ppx1. We hypothesize that these short-chain polyPs are involved in a signaling mechanism that pre-established the alkaline-resistant state of CRN/PPN2 cells.

Of multiple polyP-hydrolyzing enzymes of yeast, Ppn1 and Ppn2 are similar in endopolyphophatase activity and vacuolar localization [18]. Yet Ppn1 or Ppn2 overexpression differently affect the adaptive capabilities of yeast cells, likely due to differences in the cellular short-chain polyP abundance [18] in the respective strains. Ppn1 overexpression improves the manganese resistance [67], but only slightly contributes to adaptation to alkali [18], while Ppn2 largely improves the adaptation to alkali, but does not affect manganese resistance. It is not improbable that the adaptation processes are controlled by other not yet identified substrates of these enzymes [75,76]. Finally, while we did not explore the possible effect of Ppn2 overexpression on the dephosphorylation of polyP-phosphorylated proteins [12], we cannot rule out that these alterations could contribute to the unique transcriptomic profile of CRN/PPN2.

All in all, our CRN/PPN2 model allowed us to identify the crucial role of Ppn2 for yeast growth in the alkaline medium and reveal the functional importance of several poorly annotated genes.

## Data Availability

The RNA-Seq data are deposited in GEO under accession number GSE212193.

## Supplementary Materials

The following supporting information can be downloaded at: https://www.mdpi.com/article/10.3390/jof10110797/s1, Figure S1: The electropherogram of polyP fractions; Figure S2: The effect of alkali concentration on yeast culture density; Figure S3: Comparison of transcriptome changes in short-term alkali stress and continuous growth in mild alkaline conditions; Figure S4: Cell counts of yeast cultivated in control YPD and in YPD supplemented with 40 mM KOH; Table S1: Library sizes, gene-level read counts, results of differential expression analysis, and gene ontology enrichment; Table S2: Raw data used to plot Figure 1a and Figure S2.
