# Supplementary material for: Ppn2 Polyphosphatase Improves the Ability of S. cerevisiae to Grow in Mild Alkaline Medium
Source: J Fungi (Basel). 2024 Nov 16;10(11):797. doi: 10.3390/jof10110797 (PMC11595888; doi:10.3390/jof10110797)
Supplement: Supplementary file 1 [file jof-10-00797-s001.zip › Supplementary Figures_ver2.pdf]

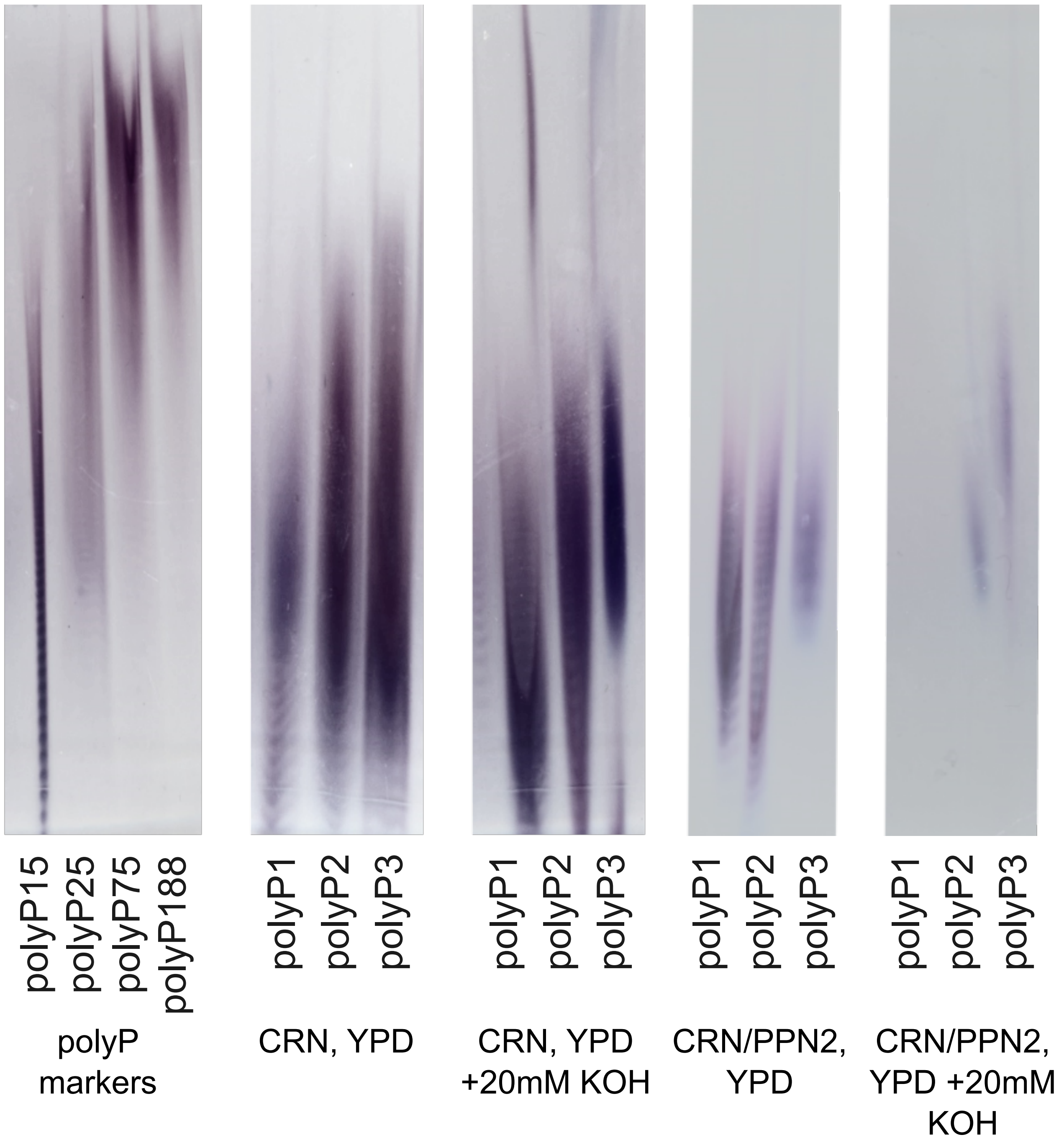

Supplementary Figure S1. The electrophoregram of polyP fractions of different chain lengths.
The polyP fractions (polyP1, polyP2, polyP3) were obtained from the cells of CRN and CRN/PPN2 strains grown in YPD for 16 h or in YPD supplemented with 20 mM KOH for 40 and 25 h, respectively. The samples with equal amounts of polyP measured by the total phosphorus content were used for electrophoresis in 24% polyacrylamide gels with 7 M urea; commercial polyP15, polyP25, and polyP75 (Sigma-Aldrich) and polyP188 (Monsanto) were used as the chain length markers (the numbers indicate the average amount of phosphate residues in the polyP chain). PolyPs were visualized by staining the gels with the toluidine blue.

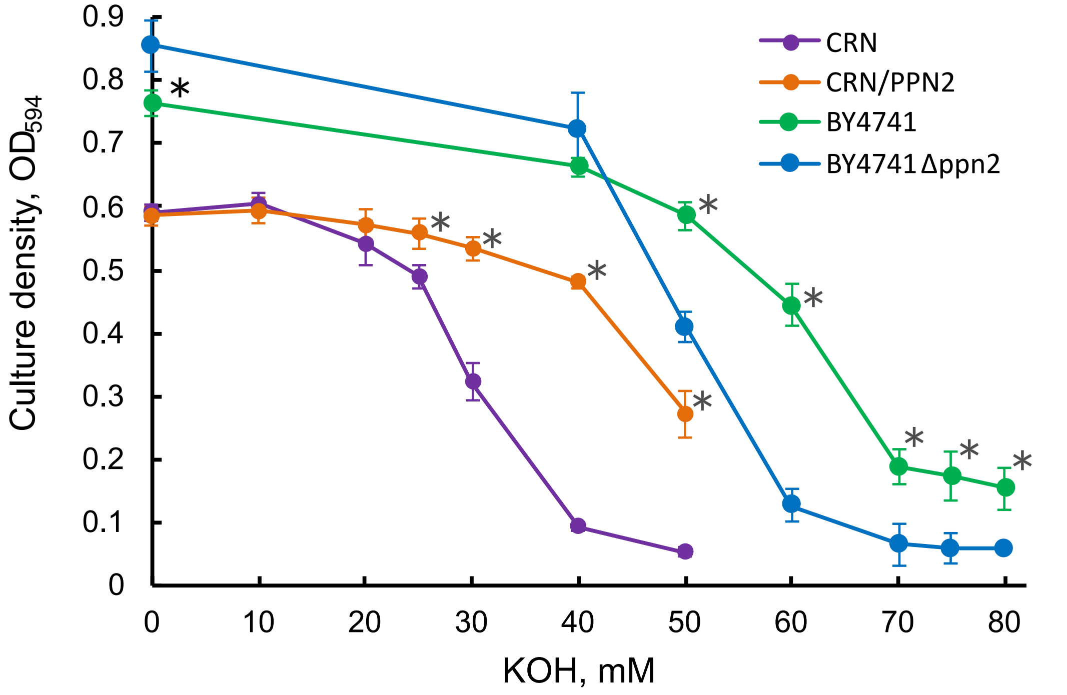

Supplementary Figure S2. The effect of alkali concentration on yeast culture density.

The effect of alkali concentration on yeast culture density, cultivation for 24h in immunoplate in YPD supplemented with varying concentrations of KOH. The culture density in 96-well plates was measured at 594 nm. Whiskers denote s.d. *$p < 0.05$, two-tailed t-test, CRN/PPN2 vs CRN, BY4741Δppn2 vs BY4741.

CRN

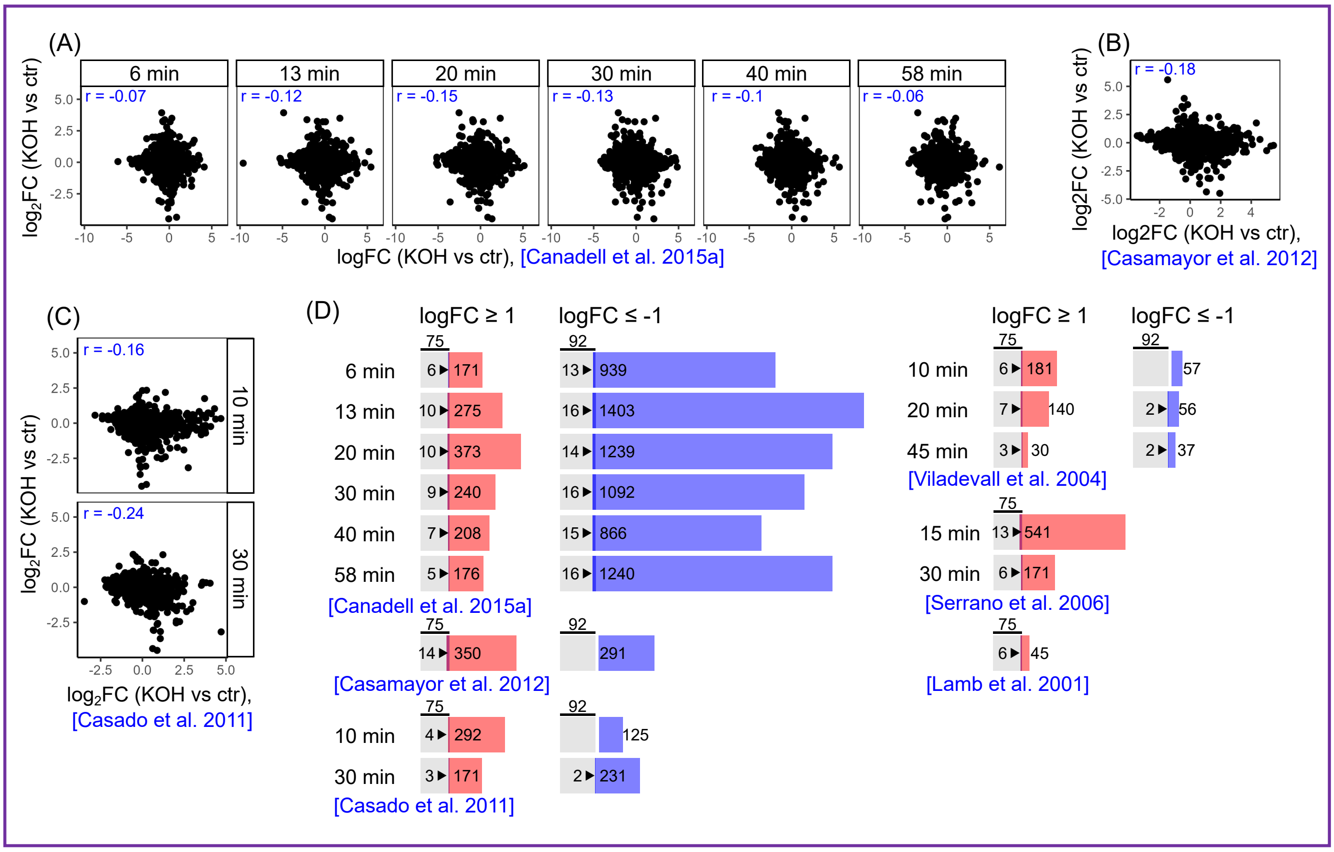

CRN/PPN2

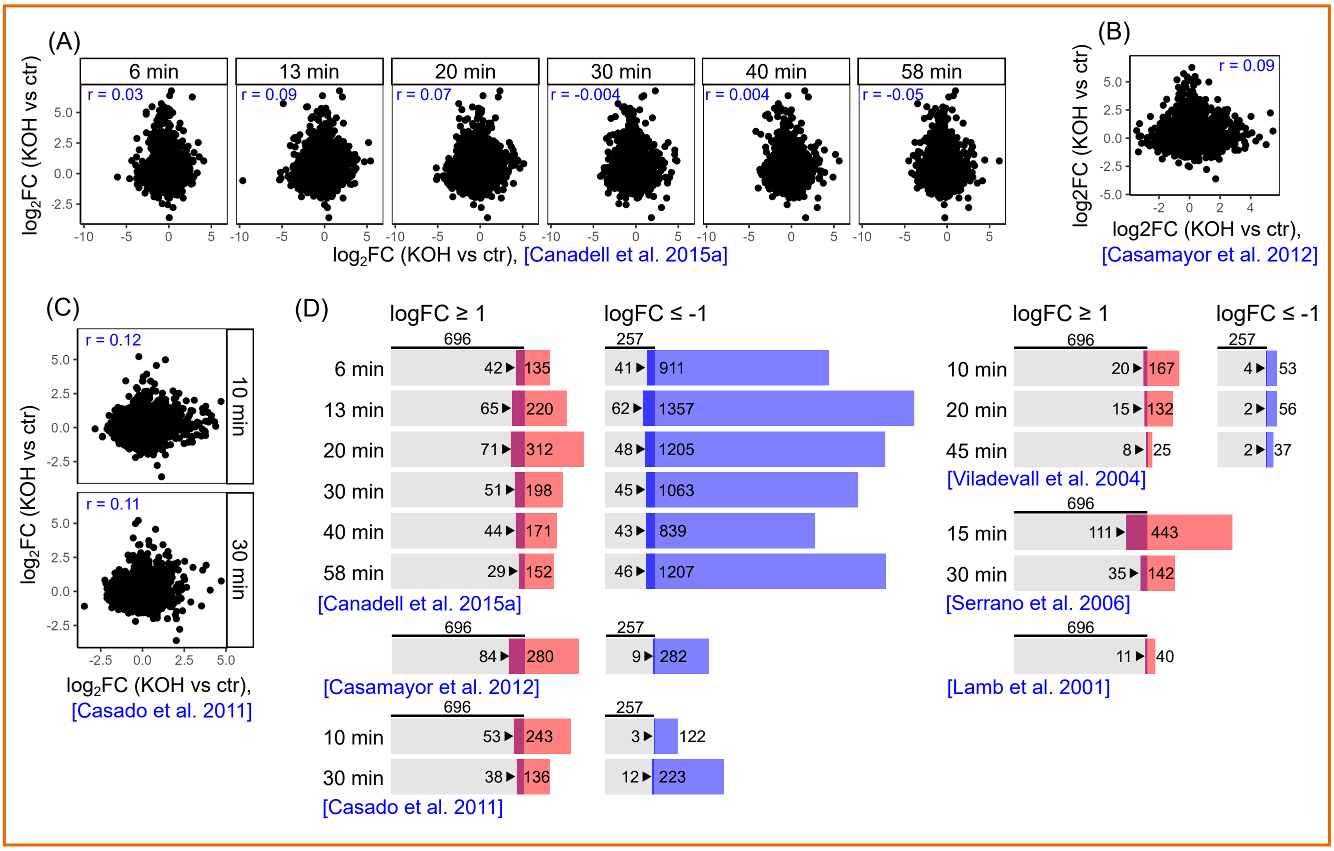

Supplementary Figure S3. Comparison of transcriptome changes in short term alkali stress and continuous growth in mild alkaline conditions.
A, B, C. Correlation between gene expression changes in alkali stress and growth in mild alkaline conditions. X-axis: published data (alkali stress); Y-axis CRN (top panel) or CRN/PPN2 (bottom panel). Expression fold changes are estimated by comparing alkaline versus normal conditions. Pearson's correlation values are labeled on the plots.
D. The number of shared and unique significantly up- and down-regulated genes in alkaline versus normal conditions for CRN (upper panel) or CRN/PPN2 (lower panel) and published alkali stress data. Grey: this study, red/blue - up/down-regulated genes according to the published data.

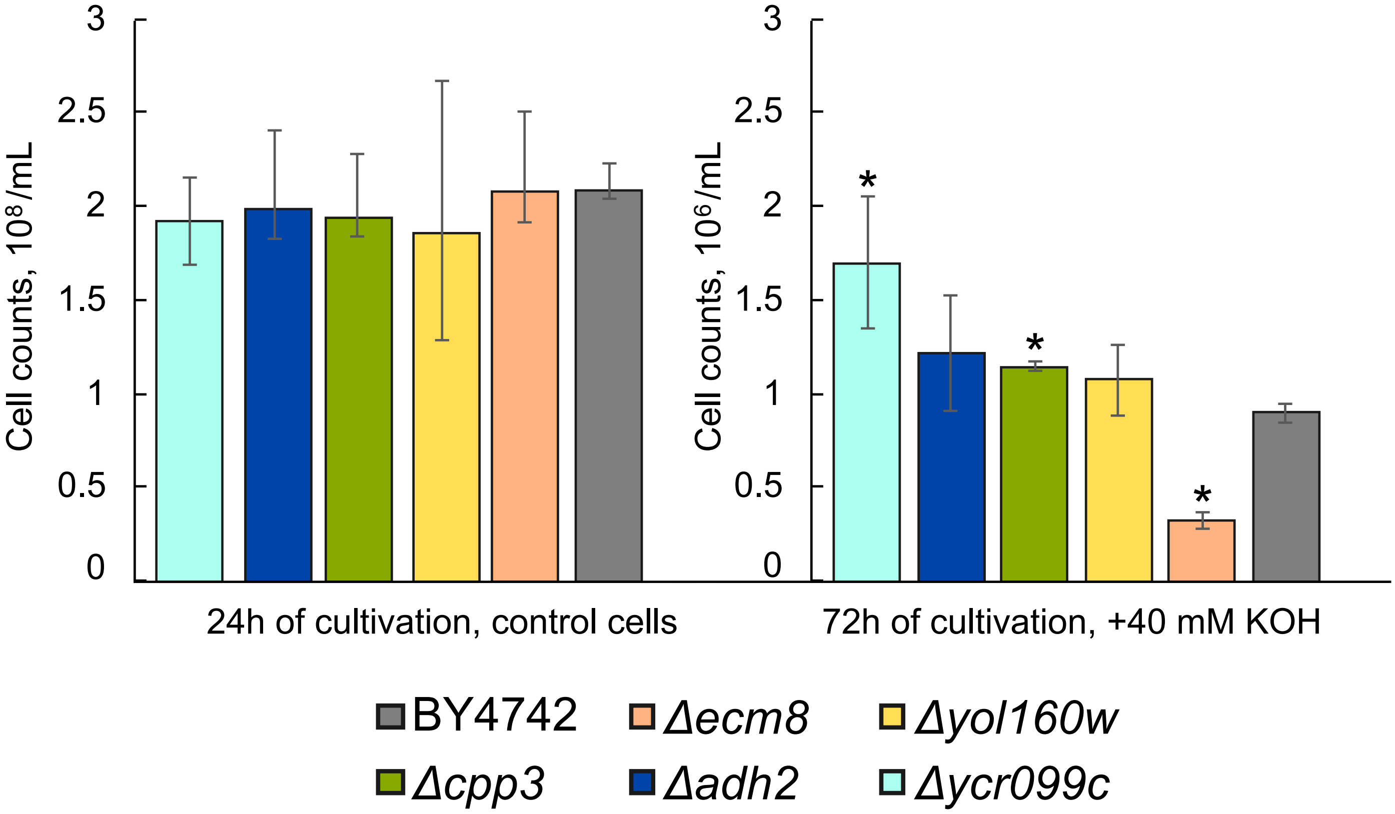

Supplementary Figure S4. Cell counts of yeast cultivated in control YPD and in YPD supplemented with 40 mM KOH, whiskers denote s.d. *$p<0.05$, two-tailed t-test against BY4742.
